# Heat transfer analysis of buoyancy opposing radiated flow of alumina nanoparticles scattered in water-based fluid past a vertical cylinder

**DOI:** 10.1038/s41598-023-37973-6

**Published:** 2023-07-03

**Authors:** Sayer Obaid Alharbi, Umair Khan, Aurang Zaib, Anuar Ishak, Zehba Raizah, Sayed M. Eldin, Ioan Pop

**Affiliations:** 1 Mathematics Department, College of Science Al-Zulfi, Majmaah University, Majmaah, 11952 Saudi Arabia; 2 Department of Mathematical Sciences, Faculty of Science and Technology, Universiti Kebangsaan Malaysia, 43600 Bangi, Selangor Malaysia; 3 Department of Mathematics and Social Sciences, Sukkur IBA University , Sukkur, Sindh 65200 Pakistan; 4 Department of Mathematical Sciences, Federal Urdu University of Arts, Science and Technology, Gulshan-e-Iqbal, Karachi, 75300 Pakistan; 5 Department of Mathematics, College of Science, King Khalid University, Abha 62529, Saudi Arabia; 6 Center of Research, Faculty of Engineering, Future University in Egypt, New Cairo, 11835 Egypt; 7 Department of Mathematics, Babeş-Bolyai University, 400084 Cluj-Napoca, Romania

**Keywords:** Applied mathematics, Mechanical engineering

## Abstract

Cooling and heating are two critical processes in the transportation and manufacturing industries. Fluid solutions containing metal nanoparticles have higher thermal conductivity than conventional fluids, allowing for more effective cooling. Thus, the current paper is a comparative exploration of the time-independent buoyancy opposing and heat transfer flow of alumina nanoparticles scattered in water as a regular fluid induced via a vertical cylinder with mutual effect of stagnation-point and radiation. Based on some reasonable assumptions, the model of nonlinear equations is developed and then tackled numerically employing the built-in bvp4c MATLAB solver. The impacts of assorted control parameters on gradients are investigated. The outcomes divulge that the aspect of friction factor and heat transport upsurge by incorporating alumina nanoparticles. The involvement of the radiation parameter shows an increasing tendency in the heat transfer rate, resulting in an enhancement in thermal flow efficacy. In addition, the temperature distribution uplifts due to radiation and curvature parameters. It is discerned that the branch of dual outcomes exists in the opposing flow case. Moreover, for higher values of the nanoparticle volume fraction, the reduced shear stress and the reduced heat transfer rate increased respectively by almost 1.30% and 0.0031% for the solution of the first branch, while nearly 1.24%, and 3.13% for the lower branch solution.

## Introduction

The analysis of nanofluids is one of the most demanding research areas due to the extensive range of applications in various industries and engineering fields. Because of their tiny size and large precise area, nanofluids have a high thermal conductivity, which aids in long-term stability and minimal blockage in a variety of physical phenomena like grinding, electronics refrigeration, peristaltic pumping utilized in treatments of diabetes, machining, and so on. Nanofluid is utilized as a coolant in industrial applications. Nanofluids can be exploited in a variety of many other applications owing to their novel heat transfer properties. Nanoparticles of metals such as gold, copper, silver, and aluminum, as well as metal oxides such as titanium oxide, alumina, and copper oxide, are employed in regular fluids like oil, ethylene glycol, and water (that have poor conductivities) to form nanofluids. In addition, alumina (Al_2_O_3_) nanoparticle is a type of metal-oxide that has a variety of applications due to their unique structural and physicochemical properties, like wear resistance, drug delivery, aqueous dispersion, coating of metal surface, etc. Choi and Eastman^1^ investigated the heat transfer aspects of nanofluids, which are liquid-particle colloidal dispersions. Later on, Khan and Pop^2^ extended the concept of nanofluid by considering the flow past a stretching sheet. They detected that the heat transport rate declines due to each dimensionless parameter. The concept of BL in nanofluid (NF) flow utilizing Ag and Cu nanoparticles was developed by Vajravelu et al.^3^. They found that the width of the boundary layer shrinks more in the case of water-based Ag compared to water-based Cu nanofluids. Makinde and Aziz^4^ studied the behavior of flow induced by nanofluids from a stretching sheet whilst accounting for the convective boundary condition. They showed that the Lewis number impact on the fluid temperature is the smallest. The performance of thermal buoyancy by incorporating nanomaterials toward a continuous permeable stretchable sheet along with slip and heat absorption/generation was scrutinized by Das^5^. Bachok et al.^6^ looked into the flow problem of unsteady adjacent to a stagnation-point incorporating nano liquid. They presented dual solutions for the decelerating flow. Uddin and Harmand^7^ examined the time-dependent flow of nanofluid across a vertical surface of the plate embedded in a porous media with free convection. They noticed that the rate of heat transfer (RHT) initially increases and then starting to decrease due to particle concentration. The steady and unsteady flows past a moving sheet with nanofluid in a constant external free stream were examined by Roşca and Pop^8^. They executed the analysis of temporal stability to check the physically realizable (stable) solution and pragmatic that the first solution is stable. Das^9^ inspected the BLF past an irregular porous stretchable sheet under the consideration of tiny nanoparticles with combined slip effects. He showed that the nanoparticle concentration uplifts due to the slip parameter. Reddy and Chamkha^10^ investigated the Soret (SR) and Dufour (DU) impacts on Lorentz forces flow in porous media (PMA) caused by water-based TiO_2_ and Al_2_O_3_ nanoparticles. They monitored a considerable improvement in heat transfer due to the presence of nanoparticles. Uddin et al.^11^ studied the impact of heat generation/absorption on the magneto flow of nanofluids through a rotatable permeable disk. They determined that nanoparticles with small sizes, greater heat absorption, and suction speed up the HT process. The characteristics of the heat transport phenomenon for the forced kind convective nanoparticles flow from a movable sheet with a heat source/sink embedded in a PMA were studied by Ghosh and Mukhopadhyay^12^. They discovered double outcomes when the free-stream and plate travel in reverse directions. Waini et al.^13^ examined the SR and DU impressions on the nanofluid flow past a slim movable needle through the Tiwari and Das model and presented binary outcomes for a single value of a parameter. In the presence of nanoparticles, it was found that the UBS of the friction factor and the HT rises while the coefficient of mass transfer falls. The impact of Lorentz forces on a cross 3D flow of streamwise direction via incorporating nanofluid using Koo–Kleinstreuer–Lee (KKL) correlation was inspected by Khan et al.^14^. It was found that the rate of mass transfer drops but the rate of heat transfer augments due to the Soret number. Uddin et al.^15^ scrutinized the impact of the magnetic field on the stagnation-point flow of nanofluid with heat transfer from a stretchable/shrinkable sheet and found dual solutions by utilizing an innovative Metaheuristic approach. Khan et al.^16^ explored the bio-convection stimulus through directions of streamwise and cross-flow cooperating nanofluid and reported dual solutions existence. Reddy and Goud^17^ explored the rule of radiation on 2D flow toward a SP induced by nanofluid across a stretchable cylinder. They observed that the temperature and the profile of nanoparticle fractions improve in response to rises the influences of the radiation parameter. Asogwa et al.^18^ examined the features of EMHD on the radiative flow of Casson nanofluid through a reactive stretchable sheet. They have perceived that the gradients increase as the modified Hartmann number rises. Goud et al.^19^ inspected the impact of radiation and Joule heating on the magneto flow of nanofluid across an exponentially stretched sheet with a medium of thermal stratified. With increasing values of the Eckert number, the TTBL (thickness of the thermal boundary-layer) is increased as a result of frictional heating. More about the significance of nanofluids can be observed in recent articles^20–22^ with different aspects.

The feature of radiation is among the most significant procedures in the movement of fluid flow and heat during a thermal system of a lofty temperature. The effect of radiation is a crucial tool for managing excessive heat emissions that has a wide variety of industrial applications. Thermal radiation has a significant impact on the structure of high-quality equipment, missiles, nuclear power plants, turbines of gas, satellites, and a wide range of complex systems of conversion mentioned in their studies^23–26^. Madhu et al.^27^ probed the time-dependent flow in non-Newtonian nanofluids by taking radiation and magnetic effects into account. They explored that the friction factor decreases in the existence of magnetic, unsteady, and Maxwell parameters. The impact of thermal radiation through a horizontal infinite surface induced by non-Newtonian fluid was calculated by Jamshed et al.^28^. In his study, the most striking finding is that the water-based copper nanofluid was found to be a better thermal conductor compared to titanium nanofluid. Yanala et al.^29^ scrutinized the slip and ramped temperature mechanisms on the transient flow past a vertical infinite plate with radiation and chemical reaction effects. A strong flow is created near the plate due to buoyancy and radiation effects, which are amplified by slip. Jamaluddin et al.^30^ analyzed the rule of radiative phenomenon on buoyancy flow near a SP induced by Cross fluid past a porous shrinkable sheet. They observed that the range of existence for dual solutions is greatly influenced by factors such as the mass transpiration, the Prandtl number, and the Weissenberg number. Goud et al.^31^ discussed the Dufour effect on the dissipative unsteady flow of Casson fluid across a laminated porous vertical laminate with a chemical reaction. The features of radiation and chemical reaction on the dissipative flow past a vertical infinite plate in a porous media with magnetic and Soret effects were analyzed by Goud et al.^32^. They found that the heat transfer rate declines as Eckert rises, while the contradictory pattern is being distinguished for radiation.

The transport features inside the region of stagnation, for instance, the polymer productivity and extrusion process are influential in the modern industry and need ongoing enhancement to preserve a high standard of quality^33,34^. As a result, the subject has piqued the researcher’s interest in the present decade. The classic two-dimensional (2D) SP flow problem was first discussed by Hiemenz^35^ and Homann^36^. Since that time, several researchers have conducted several investigations on stagnation point flow in a variety of flow systems. Kumari and Nath^37^ utilized the theory of boundary layer and the finite difference technique to simulate mixed convective SPF induced by non-Newtonian fluids (NNFs). They noticed that the buoyancy and magnetic parameters increase the gradient of surface velocity and heat transfer. The steady 2D flow near an SP simulated with nanomaterials past a stretchable/shrinkable sheet moves in its plane was extended by Bachok et al.^38^. They reported that the Cu nanoparticles outperformed the other nanoparticles in terms of the friction factor and heat transfer. Awaludin et al.^39^ performed the stability analysis for the stagnation point flow (SPF) through a linearly shrinkable or stretchable sheet and presented more than one solution. Halima et al.^40^ inspected the flow of stagnation point simulated with non-Newtonian fluid through a slippery stretching sheet with nanofluid subject to passive and active nanoparticle controls. It was discovered that due to a stagnant point, the ability of heat transport phenomena improved in both active and passive controls. The entropy generation on the SPF of a NNF suspended by nanoparticles through a moving surface with activation energy was discovered by Zaib et al.^41^. Lately, Zainal et al.^42^ investigated the SPF of a nanofluid past a stretchable sheet induced by non-Newtonian fluid and observed dual solutions. To verify the dependability of the solutions, a stability study was performed.

Many researchers are concerned with the investigation of fluid flow past a vertical cylinder. Several research papers examined the fluid flow through a moving/static cylinder and discussed the interesting aspects of flow models. Wang^43^ modeled and investigated the motion of the fluid problem over a stretchable cylinder. Ishak et al.^44^ reconfigured and adorned the problem by incorporating supplementary effects of heat transfer. These estimates were taken for cylinders that are homogeneously porous and stretchable. They observed that if there is no forceful infusion, water cools more effectively than air does. More specifically, the chronological work of Ishak and Nazar^45^ is incorporated here, where they asserted the motion of fluid along a stretchable cylinder. Wang and Ng^46^ investigated the slip influence on the fluid flow from a stretchable cylinder at the fluid–solid interface. They found that the slip parameter declines the shear stress and velocities. The HT features and dynamics of fluid flow were inspected by Gorla and Bhattacharyya^47^. They discussed the flow properties through a shrinkable permeable cylinder. They discovered that the HT is augmented owing to curvature and suction parameters. Majeed et al.^48^ explored the flow with heat transport past a stretchable cylinder by assuming partial slip and heat-flux conditions and utilized the Chebyshev spectral Newton iterative method to find the iterative solution. The viscous flow past a stretchable (shrinkable) permeable cylinder having an irregular radius was inspected by Ali et al.^49^. Reddy et al.^50^ examined the impact of entropy on the radiative transient flow with mass and heat transfer induced by a couple-stress fluid across a vertical cylinder with magnetic effect. The outcome shows that magnetic and radiation parameters decrease and ultimately increase the entropy generation. The supercritical free convective flow of couple stress and Newtonian fluids around an isothermal cylinder was addressed by Basha et al.^51^. The current computational work demonstrates that a supercritical Newtonian fluid has transient and steady-state velocity fields that are significantly higher than coupling stress fluid. Waini et al.^52^ employed binary hybrid nanomaterials to calculate the HT and liquid flow by employing the heat flux condition and found multiple solutions. Palaiah et al^53^ calculated the impression of dissipation on radiative buoyancy flow of couple-stress fluids across a vertical cylinder in a porous media with chemical reaction and magnetic effects. Ahmed et al.^54^ explored Dufour and Soret effects on the radiative flow of third-grade fluid past a stretchable cylinder. They noticed that the fields of concentration and thermal in the area of BL surrounding the cylinder are helped by the concurrent increase in Dufour and Soret numbers.

Motivated by the above literature, the current exploration analyzed the influence of radiation on the mixed convective flow towards a stagnation-point through a vertical cylinder is discussed. Different from the previous studies, the water-based alumina nanofluid near a stagnation-point is incorporated past a vertical cylinder with buoyancy effects, where the double solutions are presented. This kind of work has not been reported yet. Similarity variables play an important role in reducing the leading equations into ordinary nonlinear equations and then employing the built-in bvp4c MATLAB solver to get the numerical outcomes.

### Description of the model

**Figure 1:**
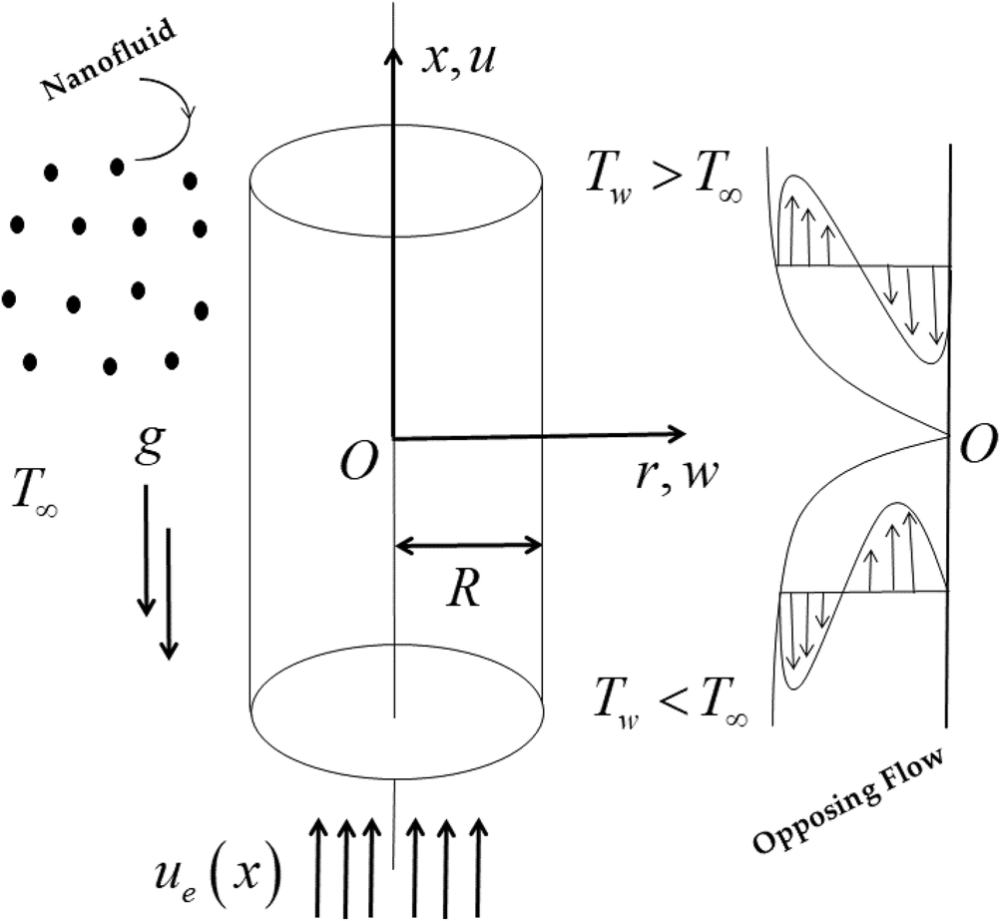
The opposing flow model and coordinate system.

Consider the steady momentum and heat transfer mixed convective flow of a stagnation point comprising alumina nanoparticles past an upright cylinder of radius $R$, as illustrated in Fig. 1. In addition, $\left( {x,\varphi ,r} \right)$ are the proposed working Cartesian cylindrical coordinates, in which, the initial $x -$ axis coordinate is measured for the way straight-up and the respective $r -$ axis reflects the considered horizontal direction, and the motion of the flow occupies the domain $r \ge 0$. The flow being axially symmetric, we have $\frac{\partial }{\partial \varphi } = 0$ for all variables. Further, suppose that the external flow or far-field velocity is denoted by $u_{e} \left( x \right) = U_{\infty } \left( \frac{x}{R} \right)$ and the variable surface temperature of the cylinder is signified by $T_{w} \left( x \right) = T_{\infty } + T_{0} \left( \frac{x}{R} \right)$, where $U_{\infty }$ is the characteristic velocity, $T_{0} < 0$ (opposing flow) is called the characteristic temperature of the base nanofluid, while the free stream temperature (base nanofluid) is symbolized by $T_{\infty }$. The term radiation heat flux $q_{r}$ is also examined in this model. The nanofluid is prepared of water-based (H_2_O) containing the type of nanomaterials, namely, alumina (Al_2_O_3_). However, the carrier-based water fluid and the posited scattered alumina nanoparticles are in thermal equilibrium (TEM). Further, the given nanoparticles have a uniform shape, and size in the TEM state.

The governing equations of nanofluid can be expressed as follows under the boundary layer scaling and certain aforesaid presumptions (see Mukhopadhyay and Ishak^55^; Devi and Devi^56^):

1 $$\frac{\partial u}{{\partial x}} + \frac{w}{r} + \frac{\partial w}{{\partial r}} = 0,$$

2 $$u\frac{\partial u}{{\partial x}} + w\frac{\partial u}{{\partial r}} - u_{e} \frac{{du_{e} }}{dx} = \frac{{\mu_{nf} }}{{\rho_{nf} }}\left( {\frac{{\partial^{2} u}}{{\partial r^{2} }} + \frac{1}{r}\frac{\partial u}{{\partial r}}} \right) + \frac{g}{{\rho_{nf} }}\left( {\rho \beta_{T} } \right)_{nf} \left( {T - T_{\infty } } \right),$$

3 $$u\frac{\partial T}{{\partial x}} + w\frac{\partial T}{{\partial r}} = \frac{{k_{nf} }}{{\left( {\rho c_{p} } \right)_{nf} }}\left( {\frac{{\partial^{2} T}}{{\partial r^{2} }} + \frac{1}{r}\frac{\partial T}{{\partial r}}} \right) - \frac{1}{{\left( {\rho c_{p} } \right)_{nf} }}\frac{1}{r}\frac{\partial }{\partial r}\left( {rq_{r} } \right),$$

with the boundary conditions

4 $$\left\{ \begin{gathered} u = w = 0,T = T_{w} \left( x \right) = T_{\infty } + T_{0} \left( \frac{x}{R} \right)\,,\,\,\,\,\,\,\,\,\,\,\,{\text{at}}\,\,\,\,\,\,\,\,\,\,r = R, \hfill \\ u \to u_{e} \left( x \right) = U_{\infty } \left( \frac{x}{R} \right),T \to T_{\infty } \,\,\,\,\,\,\,\,\,\,\,\,\,\,\,\,\,\,\,\,\,{\text{as}}\,\,\,\,r \to \infty . \hfill \\ \end{gathered} \right.$$

The components of velocity along the axial and radial directions are symbolized by $u$ and $w$, respectively. Meanwhile, $T$ signifies the nanofluid temperature and $g$ denotes the acceleration owing to gravity.

Further, the absolute viscosity $\mu_{nf}$, coefficient of thermal expansion $\left( {\rho \beta_{T} } \right)_{nf}$, density $\rho_{nf}$, specific heat capacity $\left( {\rho c_{p} } \right)_{nf}$, and thermal conductivity $k_{nf}$ of the nanofluid are defined as follows (see Zaib et al.^57^, Chu et al.^58^):

5 $$\left\{ \begin{aligned} \rho_{nf} = & \phi \rho_{s} + \left( {1 - \phi } \right)\rho_{f} , \, \left( {\rho \beta_{T} } \right)_{nf} = \left( {1 - \phi } \right)\left( {\rho \beta_{T} } \right)_{f} + \phi \left( {\rho \beta_{T} } \right)_{s} , \, \mu_{nf} = \frac{{\mu_{f} }}{{\left( {1 - \phi } \right)^{2.5} }}, \\ \alpha_{nf} = & k_{nf} /\left( {\rho c_{p} } \right)_{nf} , \, \frac{{k_{nf} }}{{k_{f} }} = \frac{{\left( {k_{s} + 2k_{f} } \right) - 2\phi \left( {k_{f} - k_{s} } \right)}}{{\left( {k_{s} + 2k_{f} } \right) + \phi \left( {k_{f} - k_{s} } \right)}}, \, \left( {\rho c_{p} } \right)_{nf} = \phi \left( {\rho c_{p} } \right)_{s} + \left( {1 - \phi } \right)\left( {\rho c_{p} } \right)_{f} . \\ \end{aligned} \right\}$$

Here, $\left( {\rho c_{p} } \right)_{f}$, $\left( {\rho c_{p} } \right)_{s}$ are the heat capacitances, $\mu_{f}$ the viscosity, $k_{f}$, $k_{s}$ the thermal conductivities, $\rho_{f}$, $\rho_{s}$ densities, and $\left( {\rho \beta_{T} } \right)_{f}$, $\left( {\rho \beta_{T} } \right)_{s}$ the coefficients of thermal expansion for the base water fluid and solid nanoparticle, while $\phi$ signifies the solid nanoparticle volume fraction and their corresponding zero value reduces the nanofluid model to a common fluid. In addition, $c_{p}$ reflects the heat capacity (HC) at a uniform pressure. Also, Table 1 shows the experimentation physical aspects of alumina (Al_2_O_3_) nanomaterials and a regular fluid.

**Table 1:** The thermo-physical data of water (H_2_O) and alumina (Al_2_O_3_) nanoparticles at $T = 25 \, ^{ \circ } {\text{C}}$ (see Riahi et al.^59^).

Physical properties	Water	Al_2_O_3_
$\beta_{T} \times 10^{ - 5} \,\left( {1/{\text{K}}} \right)$	21	0.85
$k\,\left( {\text{W/mK}} \right)$	0.613	40
$\rho \,\left( {{\text{kg/m}}^{3} } \right)$	997.1	3970
$c_{p} \,\left( {{\text{J/kg}}\,{\text{K}}} \right)$	4179	765
Pr	6.2	–

Moreover, the term $q_{r}$ is defined below, which a simplified form is obtained from the Rosseland approximations (see Bataller^60^; Ishak^61^; Magyari and Pantokratoras^62^):

6 $$q_{r} = - \frac{{4\sigma^{b} }}{{3k^{b} }}\frac{{\partial T^{4} }}{\partial r},$$

where $\sigma^{b}$ and $k^{b}$ indicate the uniform SBN and MAC, respectively. Then, the higher power term $T^{4}$ is treated mathematically about the point $T_{\infty }$ to get $T^{4} \approx 4T_{\infty }^{3} T - 3T_{\infty }^{4}$ using the Taylor series and ignores the higher-order terms. With aid of this, Eq. (3) yields:

7 $$u\frac{\partial T}{{\partial x}} + w\frac{\partial T}{{\partial r}} = \frac{{k_{nf} }}{{\left( {\rho c_{p} } \right)_{nf} }}\left( {\frac{{\partial^{2} T}}{{\partial r^{2} }} + \frac{1}{r}\frac{\partial T}{{\partial r}}} \right) + \frac{{16\sigma^{b} T_{\infty }^{3} }}{{3\left( {\rho c_{p} } \right)_{nf} k^{b} }}\left( {\frac{{\partial^{2} T}}{{\partial r^{2} }} + \frac{1}{r}\frac{\partial T}{{\partial r}}} \right),$$

or

8 $$u\frac{\partial T}{{\partial x}} + w\frac{\partial T}{{\partial r}} = \frac{{\alpha_{f} }}{{\left( {\rho c_{p} } \right)_{nf} /\left( {\rho c_{p} } \right)_{f} }}\left( {\frac{{\partial^{2} T}}{{\partial r^{2} }} + \frac{1}{r}\frac{\partial T}{{\partial r}}} \right)\left( {\frac{{k_{nf} }}{{k_{f} }} + \frac{{16\sigma^{b} T_{\infty }^{3} }}{{3k^{b} k_{f} }}} \right).$$

As in Mukhopadhyay and Ishak^55^, it is suitable to announce the following requisite posited similarity transformations:

9 $$\psi = \left( {x\upsilon_{f} u_{e} R^{2} } \right)^{\frac{1}{2}} f\left( \xi \right),\theta \left( \xi \right) = \frac{{T - T_{\infty } }}{{T_{w} \left( x \right) - T_{\infty } }},\xi = \frac{{r^{2} - R^{2} }}{2R}\left( {\frac{{u_{e} }}{{x\upsilon_{f} }}} \right)^{\frac{1}{2}} ,$$

where $\psi$ is the stream function demarcated as $ru = \partial \psi /\partial r$ and $rw = - \partial \psi /\partial x$ which satisfies the continuity Eq. (1) identically. Substituting (9) into (2) and (8) gives

10 $$\frac{{\mu _{{nf}} /\mu _{f} }}{{\rho _{{nf}} /\rho _{f} }}\left[ {\left( {1 + 2\gamma \xi } \right)f''' + 2\gamma f''} \right] + ff'' + 1 - f'^{2} + \lambda \frac{{\left( {\rho \beta _{T} } \right)_{{nf}} /\left( {\rho \beta _{T} } \right)_{f} }}{{\rho _{{nf}} /\rho _{f} }}\theta = 0,$$

11 $$\frac{1}{{\Pr \left( {\rho c_{p} } \right)_{nf} /\left( {\rho c_{p} } \right)_{f} }}\left( {\frac{{k_{nf} }}{{k_{f} }} + \frac{4}{3}R_{d} } \right)\left[ {\left( {1 + 2\gamma \xi } \right)\theta ^{\prime\prime} + 2\gamma \theta ^{\prime}} \right] + \theta ^{\prime}f - \theta f^{\prime} = 0,$$

subject to the BCs

12 $$\begin{gathered} f\left( 0 \right) = f^{\prime}\left( 0 \right) = 0,\,\,\,\theta \left( 0 \right) = 1, \hfill \\ f^{\prime}\left( \xi \right) \to 1,\,\,\,\theta \left( \xi \right) \to 0\,\,\,{\text{as}}\,\,\xi \to \infty . \hfill \\ \end{gathered}$$

The occupied non-dimensional Eqs. (10) and (11) confined the following factors such as the radiation parameter $R_{d} = 4\sigma^{b} T_{\infty }^{3} /k^{b} k_{f}$, the curvature parameter $\gamma = \left( {\upsilon_{f} /U_{\infty } R} \right)^{1/2}$, the Prandtl number $\Pr = \upsilon_{f} /\alpha_{f}$, and the mixed convection parameter $\lambda = g\beta_{T} T_{0} R/U_{\infty }^{2}$. Moreover, the mixed convection is the ratio of the Grashof number to the square of the Reynolds number.

The gradients of the current problem are the shear stress $C_{f}$ (skin friction) and the local Nusselt number $Nu_{x}$, which are defined as:

13 $$C_{f} = \frac{{\mu_{nf} }}{{\rho_{f} u_{e}^{2} }}\left. {\left( {\frac{\partial u}{{\partial r}}} \right)} \right|_{r = R} ,\,\,\,\,Nu_{x} = \frac{x}{{k_{f} \left( {T_{w} \left( x \right) - T_{\infty } } \right)}}\left. {\left( {\left( { - k_{nf} \frac{\partial T}{{\partial r}}} \right) + q_{r} } \right)} \right|_{r = R} .$$

Using (9) and (13), we obtain

14 $${\text{Re}}_{x}^{1/2} C_{f} = \frac{{\mu_{nf} }}{{\mu_{f} }}f^{\prime\prime}\left( 0 \right),\,\,\,\,{\text{Re}}_{x}^{ - 1/2} Nu_{x} = - \left( {\frac{{k_{nf} }}{{k_{f} }} + \frac{4}{3}R_{d} } \right)\theta ^{\prime}\left( 0 \right),$$

where ${\text{Re}}_{x} = xu_{e} /\upsilon_{f}$ corresponds to the local Reynolds number.

### Numerical procedure

The complete solution procedure of the scheme as well as the confirmation or validity of the code is clarified in this section. The system of higher-order ODE Eqs. (10) and (11) along with BCs (12) are numerically solved via the built-in bvp4c MATLAB solver which is based on the Lobatto IIIA formula to obtain the numerical results (see Kumar et al.^63^). In this procedure, the system of higher order ODEs is reduced to the first-order ODE equations by introducing new variables. To start the procedure, let $f = A_{a}$, $f^{\prime} = A_{b}$, $f^{\prime\prime} = A_{c}$, $\theta = A_{d}$, and $\theta ^{\prime} = A_{e}$. With the assistance of these variants, the developed third and second order ODEs are converted to the following set of first-order ODEs with high nonlinearity as follows:

15 $$\frac{d}{d\xi }\left( \begin{gathered} A_{a} \hfill \\ A_{b} \hfill \\ A_{c} \hfill \\ A_{d} \hfill \\ A_{e} \hfill \\ \end{gathered} \right) = \left( \begin{gathered} A_{b} \hfill \\ A_{c} \hfill \\ \frac{{\rho_{nf} /\rho_{f} }}{{\mu_{nf} /\mu_{f} }}\frac{1}{{\left( {1 + 2\gamma \xi } \right)}}\left( {A_{b}^{2} - 2\frac{{\mu_{nf} /\mu_{f} }}{{\rho_{nf} /\rho_{f} }}\gamma A_{c} - A_{a} A_{c} - 1 - \frac{{\left( {\rho \beta_{T} } \right)_{nf} /\left( {\rho \beta_{T} } \right)_{f} }}{{\rho_{nf} /\rho_{f} }}\lambda A_{d} } \right) \hfill \\ A_{e} \hfill \\ \frac{{\Pr \left( {\rho c_{p} } \right)_{nf} /\left( {\rho c_{p} } \right)_{f} }}{{\left( {\frac{{k_{nf} }}{{k_{f} }} + \frac{4}{3}R_{d} } \right)\left( {1 + 2\gamma \xi } \right)}}\left( {A_{d} A_{b} - A_{a} A_{e} - 2\frac{{\left( {\frac{{k_{nf} }}{{k_{f} }} + \frac{4}{3}R_{d} } \right)}}{{\Pr \left( {\rho c_{p} } \right)_{nf} /\left( {\rho c_{p} } \right)_{f} }}\gamma A_{e} } \right) \hfill \\ \end{gathered} \right),$$

with boundary conditions are

16 $$\left( \begin{gathered} A_{a} \left( 0 \right) \hfill \\ A_{b} \left( 0 \right) \hfill \\ A_{b} \left( \infty \right) \hfill \\ A_{d} \left( 0 \right) \hfill \\ A_{d} \left( \infty \right) \hfill \\ \end{gathered} \right) = \left( \begin{gathered} 0 \hfill \\ 0 \hfill \\ 1 \hfill \\ 1 \hfill \\ 0 \hfill \\ \end{gathered} \right).$$

Moreover, to start the working process of computing the solution, the values of the unknown conditions are calculated, and other key prominent parameters in transformed equations are set in a manner that would provide the necessary numerical convergence. The process of initial iteration is carried out for an appropriate finite value of $\xi = \xi_{\infty } = 10$, and the result is acknowledged only when the criteria in Eq. (16) are asymptotically met. The tolerance requirement is set $10^{ - 6}$ to yield precise numerical results. The first solution is comparatively easy to find within a CPU time of 20 s, however, for the second solution, it is very difficult to get a little more time i.e., 40 s. The detailed procedure of the scheme was given in the book of Shampine et al.^64^ as well as in many other published works of literature (see Refs.^65–67^). For verification of our scheme, the values of gradients of the upper branch solutions for different values of $\gamma$ are matched with the available published work of Grosan and Pop^68^ for the limiting case when $\lambda = 0,\,\,R_{d} = 0$, $\Pr = 6.2$ and $\phi = 0$. The outcomes of the present study are completely matched with the existing work for the three distinct choices of the curvature constraint as revealed numerically in Table 2. Hence, this outstanding assessment can give us confidence that the attained unavailable results for both solution branches are accurate.

**Table 2:** Values of the reduced skin friction and reduced heat transfer for some selected values of $\gamma$ when $\lambda = 0,R_{d} = 0$ and $\phi = 0$.

$\gamma$	Grosan and Pop^68^	Current results	Grosan and Pop^68^	Current results
0.0	1.232587	1.2325857	1.573432	1.5734317
1.0	1.707622	1.7076016	2.151733	2.1517308
5.0	3.137181	3.1371607	3.837074	3.8370725

## Results and discussion

The comprehensive analysis and physical interpretation of the results for the two distinct branch solutions with influence of various parameters are presented in this section via several graphs and tables. The characteristics of the base fluid (water) and the alumina (Al_2_O_3_) nanomaterials are shown in Table 1, while the comparison for the restrictive cases is being exhibited in Table 2. Therefore, Table 3 compares the results of the numerical calculation for RSS (upper branch solution) made using bvp4c and NDSolve. The two numerical procedures used to generate the reported RSS data exhibit excellent/outstanding concordance up to three decimal places.

**Table 3:** Comparison of numerical results of RSS (UBS) by applying bvp4c and NDSolve.

$\phi$	$\gamma$	Bvp4c	NDSolve
0.025	0.05	0.2617035	0.2616311
0.030	–	0.2651161	0.2650522
0.035	–	0.2685406	0.2684111
0.025	0.0	0.2248253	0.2245178
–	0.05	0.2685406	0.2685313
–	0.10	0.3091317	0.3090974

**Figure 2:**
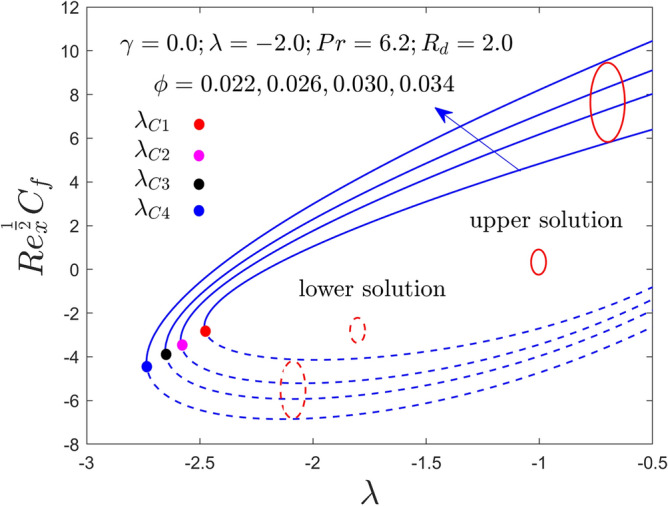
Variation of RSS with $\lambda$ for $\phi = 0.022,0.026,0.030,0.034$ and $\gamma = 0.0$ when $\lambda < 0$.

**Figure 3:**
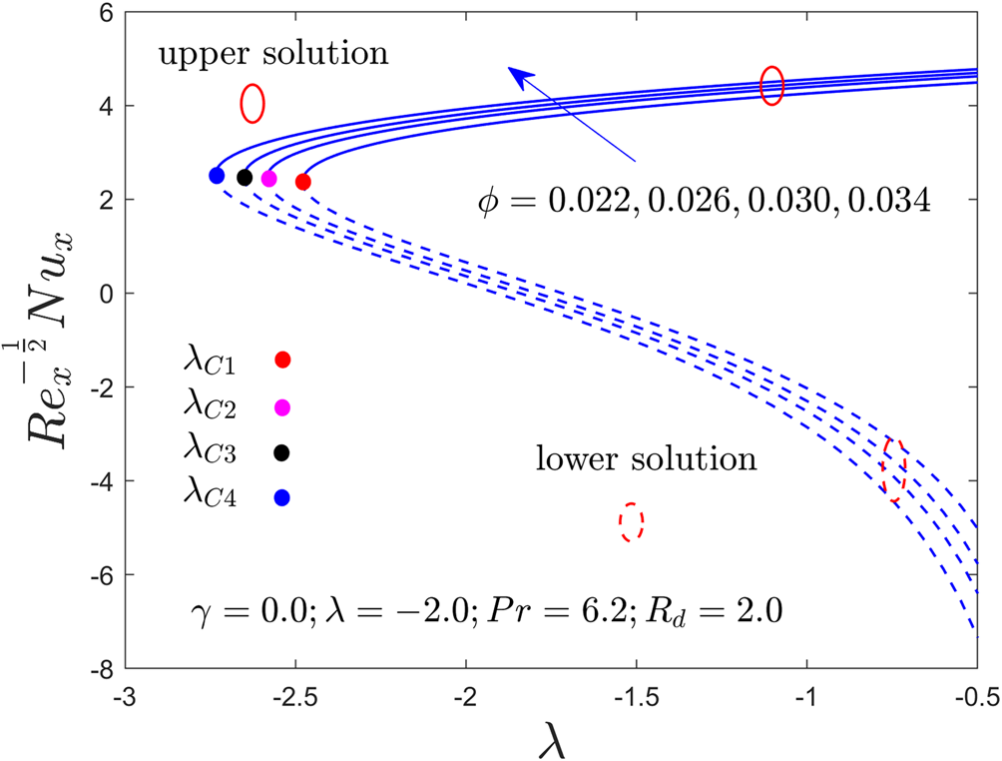
Variation of RHT with $\lambda$ for $\phi = 0.022,0.026,0.030,0.034$ and $\gamma = 0.0$ when $\lambda < 0$.

**Figure 4:**
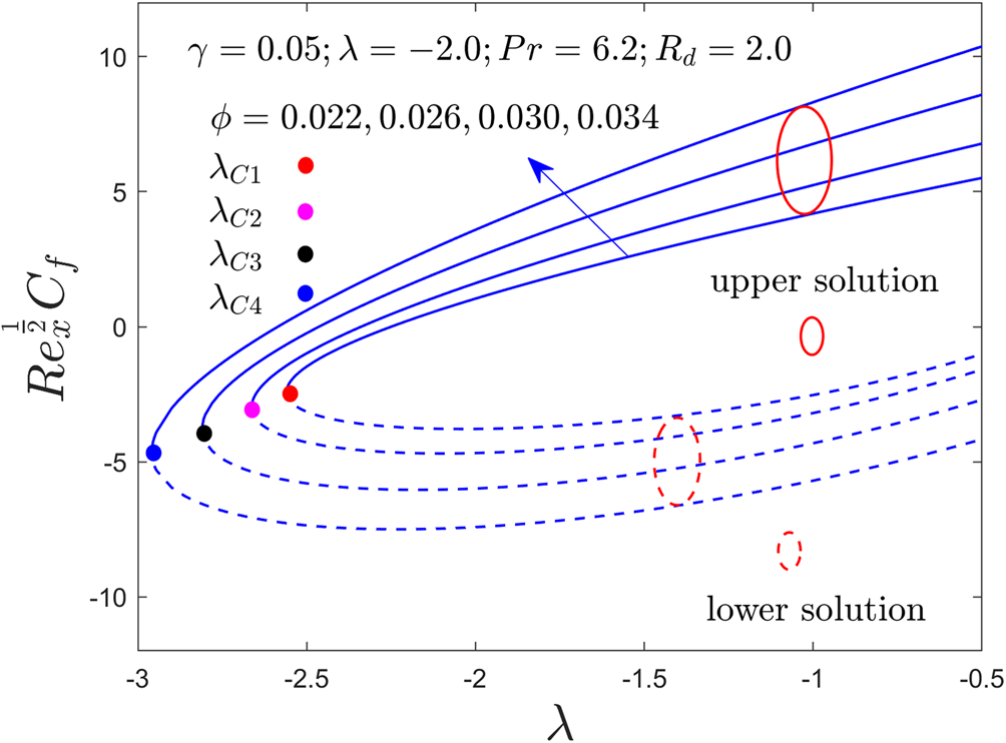
Variation of RSS with $\lambda$ for $\phi = 0.022,0.026,0.030,0.034$ and $\gamma = 0.05$ when $\lambda < 0$.

**Figure 5:**
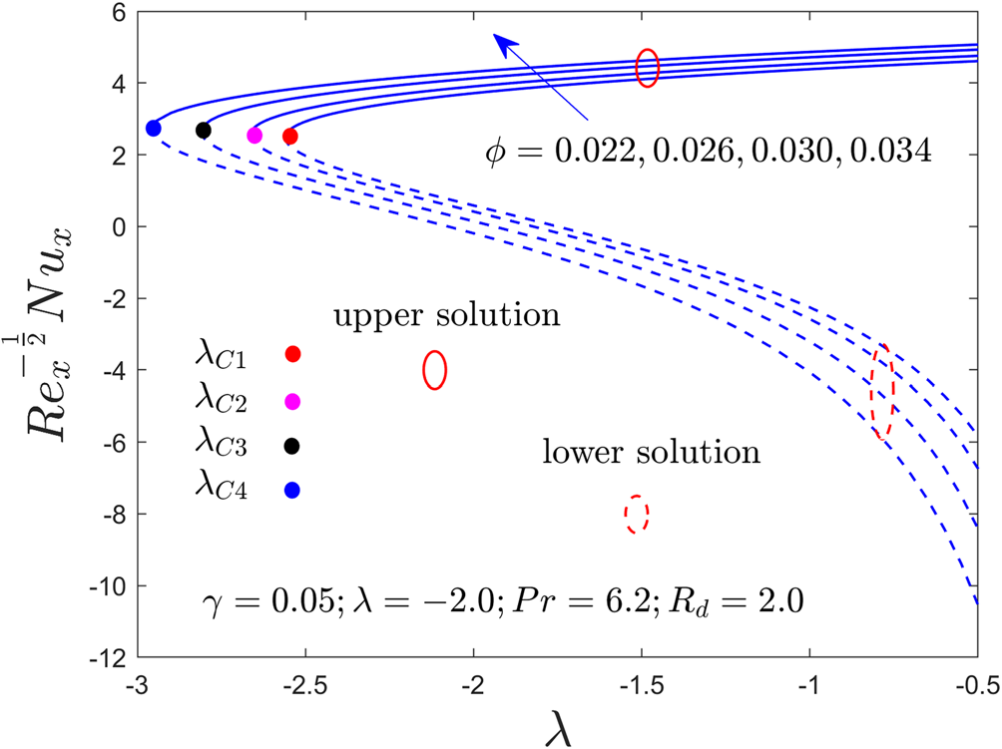
Variation of RHT with $\lambda$ for $\phi = 0.022,0.026,0.030,0.034$ and $\gamma = 0.05$ when $\lambda < 0$.

**Figure 6:**
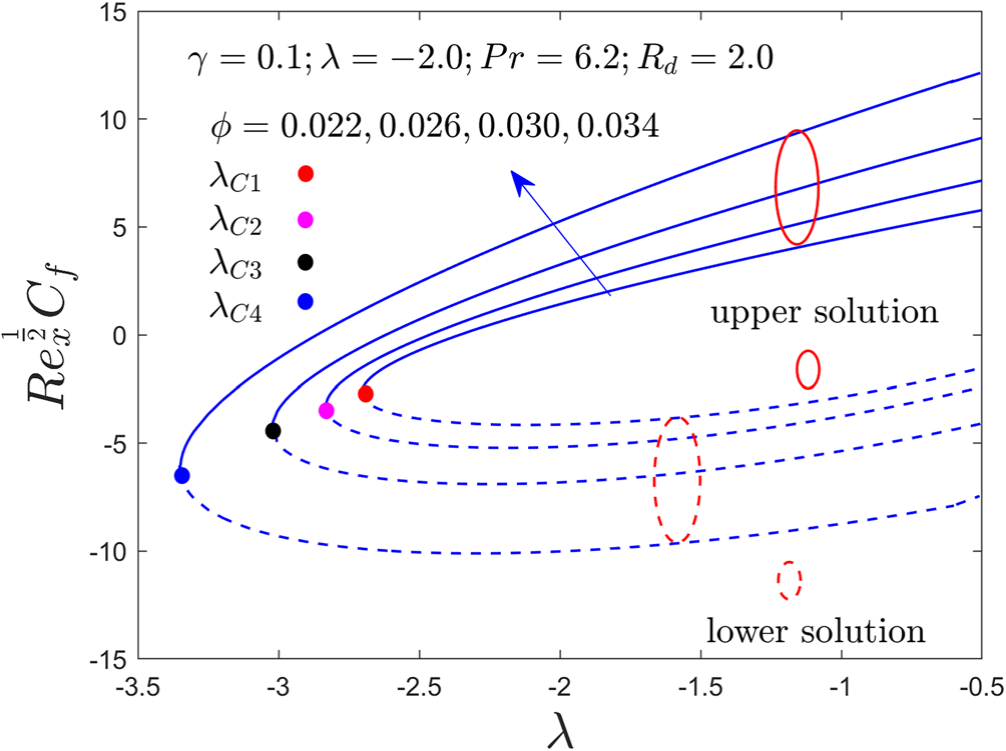
Variation of RSS with $\lambda$ for $\phi = 0.022,0.026,0.030,0.034$ and $\gamma = 0.1$ when $\lambda < 0$.

**Figure 7:**
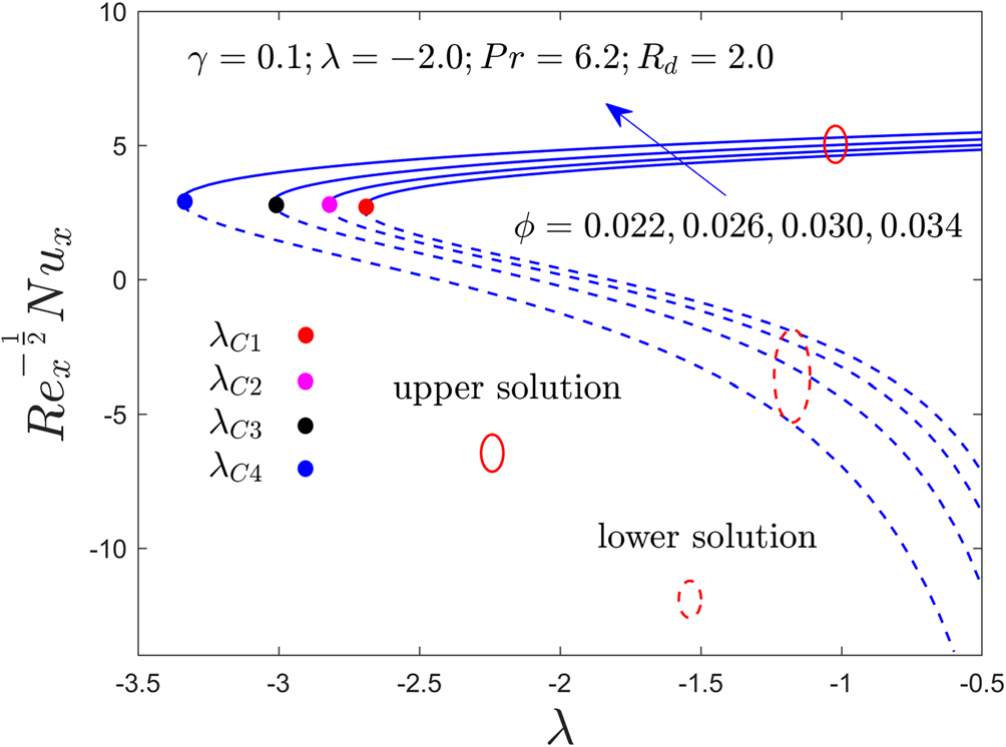
Variation of RHT with $\lambda$ for $\phi = 0.022,0.026,0.030,0.034$ and $\gamma = 0.1$ when $\lambda < 0$.

**Figure 8:**
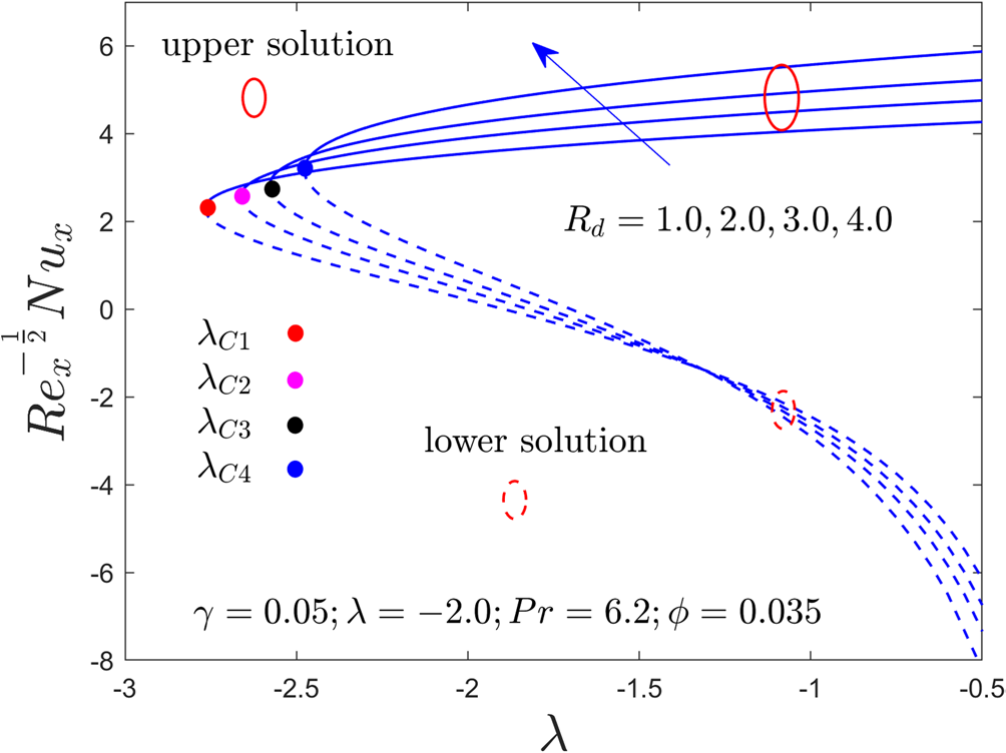
Variation of RHT with $\lambda$ for $R_{d} = 1.0,2.0,3.0,4.0$ and $\gamma = 0.05$ when $\lambda < 0$.

**Figure 9:**
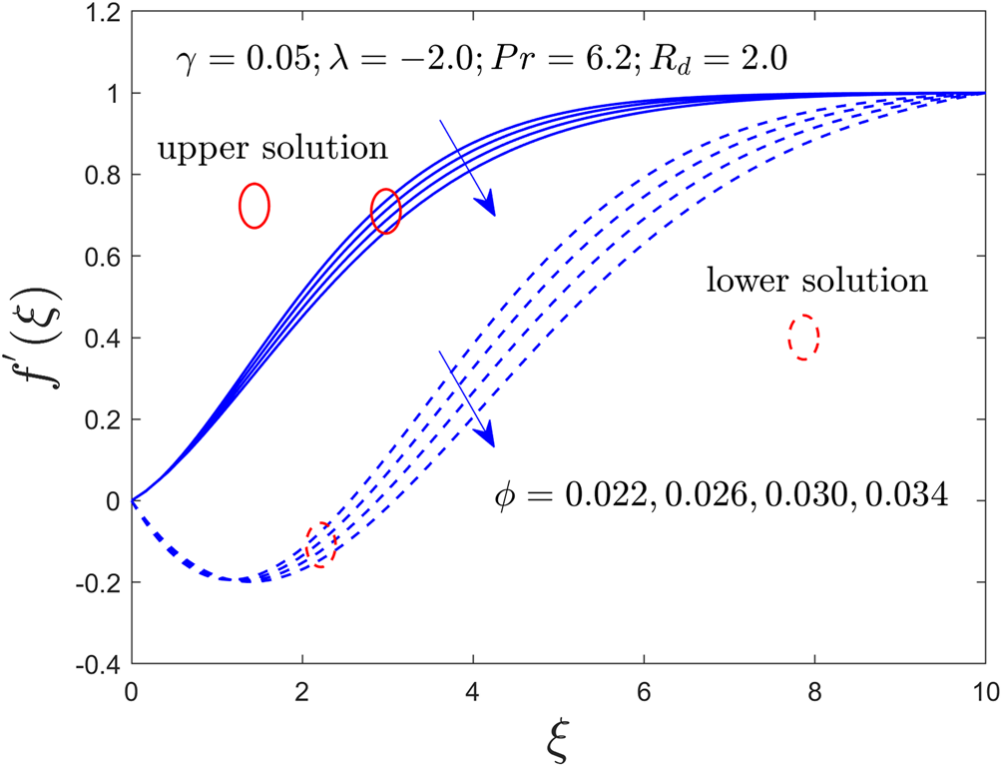
Velocity profile with $\xi$ for $\phi = 0.022,0.026,0.030,0.034$ and $\gamma = 0.05$.

**Figure 10:**
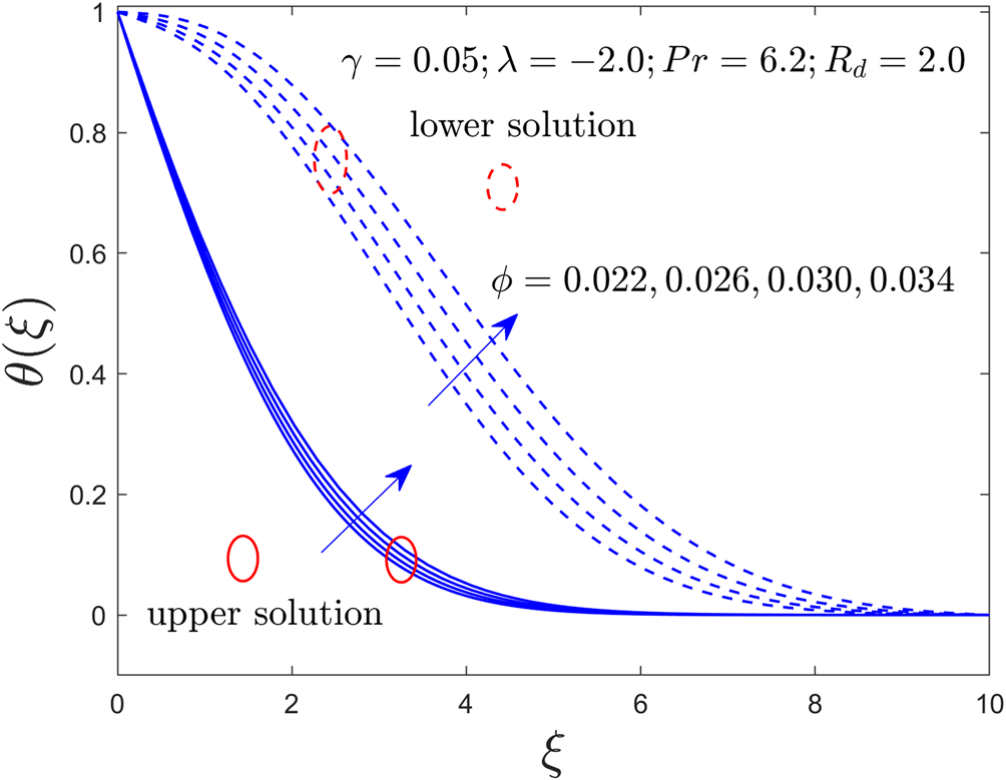
Temperature profile with $\xi$ for $\phi = 0.022,0.026,0.030,0.034$ and $\gamma = 0.05$.

**Figure 11:**
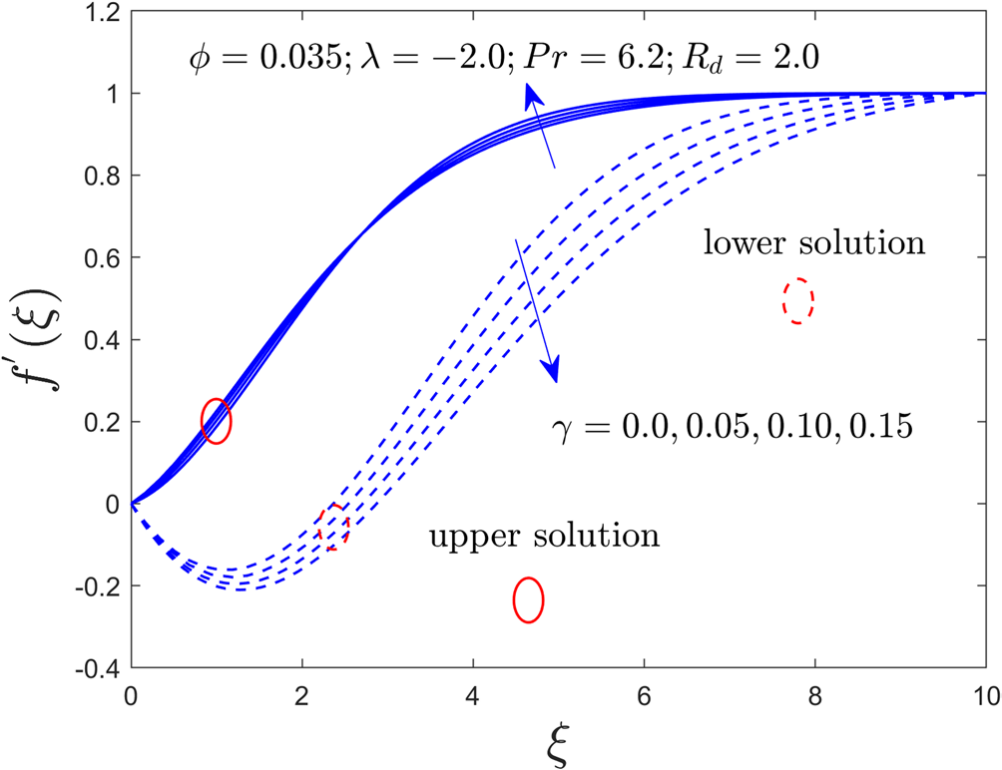
Velocity profiles versus $\xi$ for $\gamma = 0.0,0.05,0.10,0.15$.

**Figure 12:**
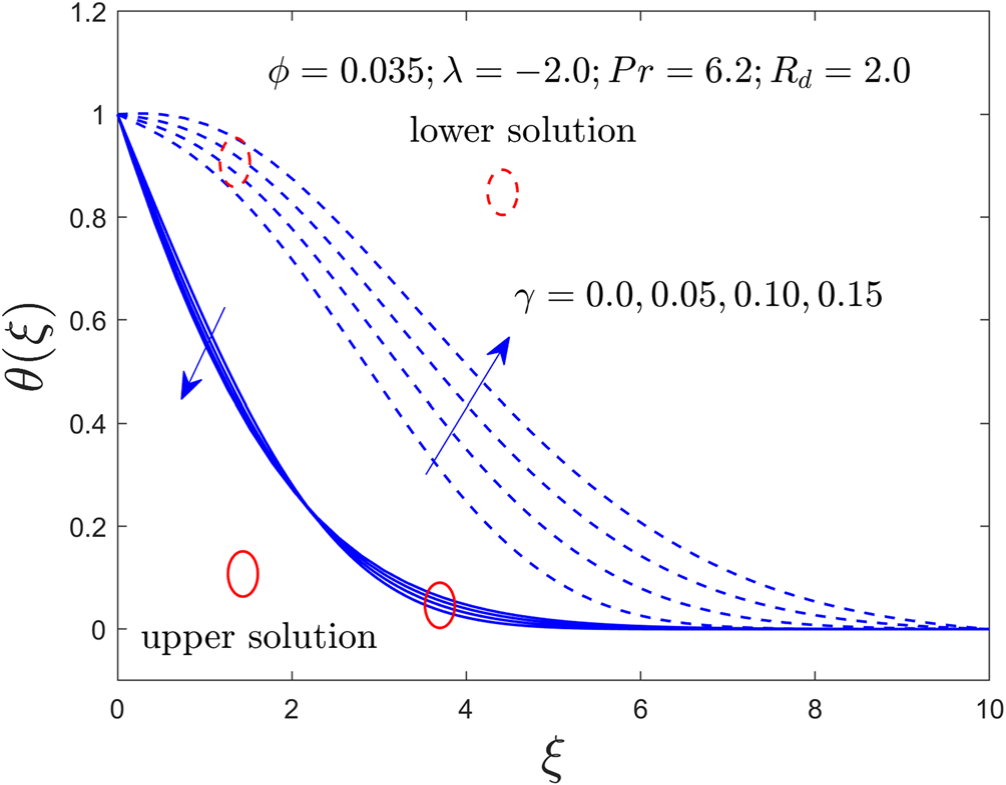
Temperature profile versus $\xi$ for $\gamma = 0.0,0.05,0.10,0.15$.

**Figure 13:**
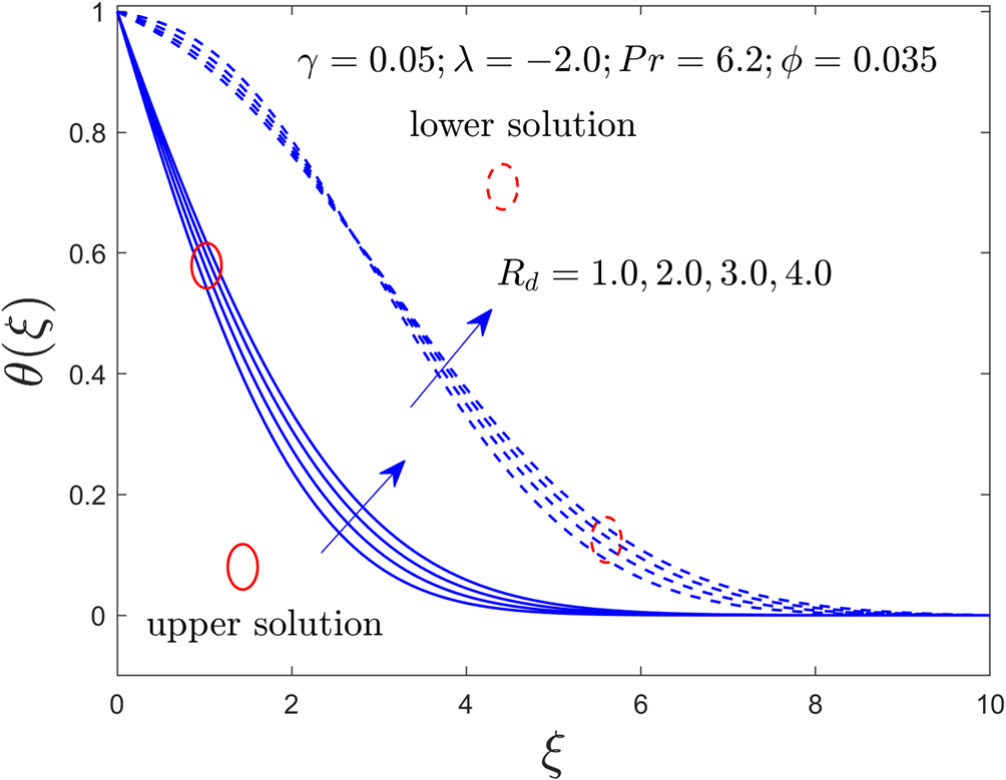
Temperature profile versus $\xi$ for $R_{d} = 1.0,2.0,3.0,4.0$ and $\gamma = 0.05$.

Further, this segment is aimed to see the stimulus of the involved factors like the curvature constraint $\gamma$, the radiation constraint $R_{d}$, the nanoparticles volume fraction $\phi$, and the mixed convection constraint $\lambda$ on velocity profile, temperature profile, heat transfer and friction factor for the stable and unstable results as graphically decorated in Figs. 2, 3, 4, 5, 6, 7, 8, 9, 10, 11, 12 and 13. In the present simulation, the following numerical values are fixed such as $\gamma = 0.05$, $R_{d} = 2.0$, $\lambda = - 2.0$ and $\phi = 0.035$ for the comprised governing parameters. Meanwhile, the Pr is taken to be $6.2$ for the considered regular fluid (water). In addition, the reduced shear stress (RSS) and reduced heat transfer (RHT) computational values for both solution branches due to distinct varying parameters are presented in Tables 4 and 5, respectively. From these results, it is exposed that the RSS upsurges for the UBS are due to the bigger value of $\phi$ and $\gamma$ while the trend of the upshots is contrary for the LBS. Oppositely, the RHT augments in both branches of outcomes with varying all three parameters. Moreover, the RSS increases by 1.30% and 1.24% in the respective UB and LB results due to the superior values of $\phi$ while it decreases by 0.77% for the LB and enhances by 19.44% for the LB with superior values of $\gamma$. On the other hand, the RHT escalates for the UB solutions in percentage-wise at around 0.0031%, 5.23%, and 11.42% owing to the higher values of $\phi$, $\gamma$ and $R_{d}$, respectively. Meanwhile, for the branch of lower solutions, the RHT enriches by 3.13%, 13.01% and 51.44% for the continuous uplifting in the values of $\phi$, $\gamma$ and $R_{d}$, respectively. The present paper reports the existence of two solutions, which are termed first and second solutions, or upper and lower solutions, based on how they appear and are shown in Fig. 2. These solutions are graphically characterized by the blue dash and blue solid lines, respectively. Meanwhile, the small solid distinct balls denote the bifurcation points.

**Table 4:** Quantitative outputs of the RSS for distinct values of the governing constraints $\phi$ and $\gamma$ when $\lambda = - 2$.

$\phi$	$\gamma$	UBS	LBS
0.025	0.05	0.2617035	− 0.6043755
0.030	–	0.2651161	− 0.6118517
0.035	–	0.2685406	− 0.6192661
0.025	0.0	0.2248253	− 0.6091188
–	0.05	0.2685406	− 0.6043755
–	0.10	0.3091317	0.0376646

**Table 5:** Quantitative outputs of the RHT for distinct values of the governing constraints $\phi$, $R_{d}$ and $\gamma$ when $\lambda = - 2$ and $\Pr = 6.2$.

$\phi$	$\gamma$	$R_{d}$	UBS	LBS
0.025	0.05	2.0	2.9566938	0.6733296
0.030	–	–	2.9567861	0.6944518
0.035	–	–	2.9569400	0.7153913
0.025	0.0	–	2.8099193	0.5958110
–	0.05	–	2.9569400	0.6733296
–	0.10	–	3.0950227	2.5696593
0.025	0.05	1.5	2.6538844	0.4446133
–	–	2.0	2.9569400	0.6733296
–	–	2.5	3.2308382	0.9181410

Figures 2, 3, 4, 5, 6 and 7 demonstrate the RSS and RHT of the Al_2_O_3_-water nanofluid for the UB and LB results against $\lambda$ with varying nanoparticles volume fraction $\phi$ and $\gamma = 0.0$, $0.05$ and $\gamma = 0.1$. These graphs display that the acquired similarity ODEs (10) and (11) with BCs (12) have possible binary outcomes for the case of BOF with varying the suitable employed parameters. Multiple solutions exist for the range of $\lambda_{C} < \lambda < 0$, no solution is possible for the range of $\lambda < \lambda_{C}$, and a single or unique output is obtained for the condition $\lambda = \lambda_{C}$. Furthermore, the minimum value of $\lambda$ is denoted by $\lambda_{C}$ while it is also dependent on the varying influential parameter values. The RSS and RHT accelerate for the UBS with the bigger value of $\phi$ and $\gamma$ while the movement of both gradients lessens for LBS. The values of $\lambda$ at the bifurcation points shown in Figs. 2 and 3 are − 2.4804, − 2.5858, − 2.6535, and − 2.7372 for the change values of $\phi$ when $\gamma = 0.0$. Similarly, (− 2.5610, − 2.6644, − 2.8120, − 2.9620) and (− 2.7047, − 2.8339, − 3.0244, − 3.3722) are the following bifurcation values (BVs) mentioned in Figs. 4, 5, 6, and 7, respectively, for the increasing value of $\phi$ when $\gamma = 0.05$ and $\gamma = 0.1$. Thus, the magnitude of the BVs $\left| {\lambda_{C} } \right|$ intensify with the magnification of $\phi$ and $\gamma$. This tendency displays that the gap of the BLR (boundary-layer thickness) declines due to the larger impression of $\phi$ and $\gamma$. Physically, the advanced alumina nanoparticles make the fluid flow more viscous, which causes the required minimum BLR to fall. However, as the volume percentage of solid nanoparticles rises, the thermal conductivity increases, and the temperature profile rises as a result. Also, the figures suggest that higher amplification of $\phi$ and $\gamma$ in the existing solution range for both branches to the similarity Eqs. (10) and (11). Between these two results, we expect that the UBS is physically acceptable and stable in the long run, while the LBS is not acceptable (unstable) as time evolves. Thus, Weidman et al.^69^ and Khan et al.^70^ for example have described the process for determining this temporal stability, therefore we will not repeat it here.

The impact of $R_{d}$ on the RHT of the Al_2_O_3_-water nanofluid for binary outcomes versus the buoyancy opposing flow is graphically illustrated in Fig. 8. The results justify that the RHT enriches for the upper solution with large values of $R_{d}$, while the behavior for the LBS is reversed, owing to greater augmentation for the selected choices of the parameter $R_{d}$. In addition, the magnitude or absolute of the BVs reduces with larger $R_{d}$. This pattern suggests that the boundary layer separation improves here with the superior impact of $R_{d}$. Physically, the effective fluid receives additional heat through the radiative heat flux. Consequently, a wider thermal boundary layer is observed.

Figures 9 and 10 describe the impacts of $\phi$ on $f^{\prime}\left( \xi \right)$ and $\theta \left( \xi \right)$ fields of the Al_2_O_3_-water nanofluid for the solid and dashed line curves, respectively. From these figures, it is noticed that the velocity declines but the curves of temperature boosted up for the UB and LB solution with superior impact on the nanoparticle volume fraction. Moreover, the separation gap for the LBS is slightly better than for the UBS. Physically, the higher concentration of the nanoparticle volume fractions creates an extra improvement in the thermal conductivity due to which the thermal boundary layer thickness and the temperature distribution escalate.

The curvature $\gamma$ and radiation $R_{d}$ parameters influence on $f^{\prime}\left( \xi \right)$ and $\theta \left( \xi \right)$ fields of the Al_2_O_3_-water nanofluid for the UB and LB outcomes are exhibited graphically in Figs. 11, 12, and 13, respectively. Noted from the output curves that the velocity $f^{\prime}\left( \xi \right)$ ascents for the UBS for higher impacts of $\gamma$ while the velocity behavior of the curves slow down for the lower branch solutions. In contrast, the temperature acts differently in both branches of the solution as compared to velocity when the rule of $\gamma$ increases. Meanwhile, the temperature distribution curves intensify for both explanations owed to bigger implementation of $R_{d}$. Generally, the higher values of $R_{d}$ boosted the thermal conductivity (TCN), and as a consequence, the behavior of the temperature and the BLR thickness rises.

## Conclusions

The idea of this work is to study the buoyancy effects of a stagnation point water-based alumina nanoparticles flow and HT aspects over a vertical cylinder with prescribed surface temperature, radiation effect, and external flow was theoretically examined. The key findings for the considered model are summarized as follows:

- Multiple (lower and upper) branch solutions are observed to survive when the buoyancy or mixed convective parameter $\lambda$ is negative [case of opposing flow or cylinder is cooled ($T_{w} \left( x \right) < T_{\infty }$)].
- The magnitude of the heat transfer and drag forces for the upper branch solutions is augmented with higher influences of the nanoparticle volume fractions, however, the trend is changed for the LBS.
- The RSS and RHT escalate owing to the solid volume fraction of nanoparticles by almost 1.30% and 0.0031% for the UBS, while for LBS, it is heightened by almost 1.24% and 3.13%, respectively.
- For the superior influence of the radiation parameter, the reduced heat transfer improves by almost 11.42% for the UBS and 51.44% for the LBS.
- The existing problem reduces to that of the special geometry (flat plate) when the effects of the curvature parameter are taken to be zero.
- Both gradients in magnitude-wise at the surface are lower for a flat plate compared to a cylinder.
- The BVs upsurge with $\phi$ but reduce due to the larger value of the radiation factor.
- The temperature augmented in the UB and LB outcomes owing to larger radiation parameter.
- For higher volume fractions of nanoparticles, the velocity curves moderate for the branch of binary outcomes but the temperature increases.

We believe that the current outcomes will offer significant information for complex issues within computer routines involving nanofluid with buoyancy force because of their several applications in processes of heat transfer, heat exchanger, solar collector, etc., and also utilize these results in experimental studies.

## Data Availability

The datasets used and/or analysed during the current study available from the corresponding author on reasonable request.

## List of symbols

$g$: Acceleration owing to gravity (m/s^2^)
$q_{r}$: Radiation heat flux
$k^{b}$: Coefficient of mean absorption
Pr: Prandtl number
$C_{f}$: Local shear stress
$$f^{\prime}\left( \xi \right)$$: Dimensionless velocity
$Nu_{x}$: Local heat transfer
$$\theta \left( \xi \right)$$: Dimensionless temperature
$R$: Radius of the cylinder (m)
$$R_{d}$$: Radiation parameter
${\text{Re}}_{x}$: Local Reynolds number
$$\left( {x,\varphi ,r} \right)$$: Cylindrical coordinates (m)
$u_{e}$: External flow velocity (m/s)
$$U_{\infty }$$: Characteristic velocity (m/s)
$T$: Temperature of the cylinder (K)
$T_{0}$: Characteristic temperature of the base nanofluid (K)
$T_{wa}$: Wall surface temperature (K)
$T_{\infty }$: Free stream temperature (K)
$\left( {u,w} \right)$: Components of velocity (m/s)

## Greek symbols

$$\gamma$$: Curvature parameter
$$\mu$$: Viscosity (kg/m s)
$\lambda$: Mixed convection parameter
$$\rho$$: Density (kg/m^3^)
$\left( {\rho \beta_{T} } \right)$: Thermal expansion coefficient (1/K)
$k$: Thermal conductivity (kg m/s^3^ K)
$\left( {\rho c_{p} } \right)$: Specific heat capacity (m^2^/s^2^ K)
$$\upsilon_{f}$$: Kinematic viscosity (m^2^/s)
$\varphi$: Solid nanoparticle volume fraction
$$\sigma^{b}$$: Stefan–Boltzmann
$$\psi$$: Stream function
$\xi$: Pseudo-similarity variable

## Subscripts

*nf*: Nanofluid
*s*: Solid nanoparticles
$$\infty$$: Ambient conditions
*f*: Base fluid
$$w$$: Wall conditions

## Superscript

*'*: Derivative with respect to $\xi$
