# Author Correction: Heat transfer analysis of buoyancy opposing radiated flow of alumina nanoparticles scattered in water-based fluid past a vertical cylinder

**DOI:** 10.1038/s41598-023-38727-0

**Published:** 2023-07-17

**Authors:** Sayer Obaid Alharbi, Umair Khan, Aurang Zaib, Anuar Ishak, Zehba Raizah, Sayed M. Eldin, Ioan Pop

**Affiliations:** 1 Mathematics Department, College of Science Al-Zulfi, Majmaah University, Majmaah, 11952 Saudi Arabia; 2 Department of Mathematical Sciences, Faculty of Science and Technology, Universiti Kebangsaan Malaysia, 43600 Bangi, Selangor Malaysia; 3 Department of Mathematics and Social Sciences, Sukkur IBA University , Sukkur, Sindh 65200 Pakistan; 4 Department of Mathematical Sciences, Federal Urdu University of Arts, Science and Technology, Gulshan-e-Iqbal, Karachi, 75300 Pakistan; 5 Department of Mathematics, College of Science, King Khalid University, Abha 62529, Saudi Arabia; 6 Center of Research, Faculty of Engineering, Future University in Egypt, New Cairo, 11835 Egypt; 7 Department of Mathematics, Babeş-Bolyai University, 400084 Cluj-Napoca, Romania

Correction to: *Scientific Reports* 10.1038/s41598-023-37973-6, published online 03 July 2023

The original version of this Article contained an error in the Acknowledgements section.

“The author (Z. Raizah) extends her appreciation to the Deanship of Scientific Research at King Khalid University, Abha, Saudi Arabia, for funding this work through the Research Group Project under Grant Number (RGP.1/334/43).”

now reads:

“The author (Z. Raizah) extends her appreciation to the Deanship of Scientific Research at King Khalid University, Abha, Saudi Arabia, for funding this work through the Research Group Project under Grant Number (RGP.1/300/44).”

The original Article has been corrected.

